# Consumption of highly processed foods in relation to overall diet quality among Japanese adults: a nationwide study

**DOI:** 10.1017/S1368980023000721

**Published:** 2023-09

**Authors:** Nana Shinozaki, Kentaro Murakami, Keiko Asakura, Shizuko Masayasu, Satoshi Sasaki

**Affiliations:** 1 Department of Social and Preventive Epidemiology, School of Public Health, The University of Tokyo, 7-3-1 Hongo, Bunkyo-ku, Tokyo 113-0033, Japan; 2 Department of Environmental and Occupational Health, School of Medicine, Toho University, Ota-ku, Tokyo, Japan; 3 Ikurien-naka, Naka-shi, Ibaraki, Japan

**Keywords:** Highly processed foods, Ultra-processed foods, Diet quality, Japan

## Abstract

**Objective:**

To (i) examine the consumption of highly processed foods (HPF) in relation to diet quality among Japanese adults and (ii) compare the results when dishes prepared away home are disaggregated into food ingredients before classification by processing levels and the results when they are not.

**Design:**

Cross-sectional analysis using 4-day dietary record data. Foods were categorised by level of processing using the framework developed by the University of North Carolina at Chapel Hill. Specifically, dishes prepared away from home were classified at both the food level (classified after disaggregation into ingredients) and dish level (classified without disaggregation). Diet quality was assessed using the Healthy Eating Index-2015 and Nutrient-Rich Food Index 9·3.

**Setting:**

Twenty areas in Japan.

**Participants:**

Adults aged 20–69 years (*n* 388).

**Results:**

Energy contribution of HPF was higher when dishes prepared away from home were classified at dish level than food level (48·3 % *v*. 32·9 %, *P* < 0·0001). Regardless of the classification method, cereals and starchy foods were the top food groups contributing to total energy intake from HPF. After adjusting for potential confounders, participants in higher tertiles of the energy contribution of HPF had lower total scores for Healthy Eating Index-2015 and Nutrient-Rich Food Index 9·3 (*P* for trend ≤ 0·007 for all), irrespective of the food- or dish-level classification.

**Conclusions:**

HPF accounted for at least one-third of energy intake of Japanese adults. Regardless of the classification methods for dishes prepared away from home, higher consumption of HPF was associated with lower diet quality.

Highly/ultra-processed foods (HPF), defined as multi-ingredient industrially formulated mixtures^(1)^, are increasingly consumed in many countries^(2)^. In recent years, many epidemiological studies have focussed on the association between HPF consumption and health outcomes. Meta-analyses have shown positive associations between HPF consumption and overweight and obesity, CVD, cerebrovascular disease, metabolic syndrome, depression and mortality^(3,4)^. Compared with non-HPF, HPF have an unhealthy nutrient profile with a higher energy density and higher contents of total fats, saturated fats, trans fats, free sugars and Na, as well as lower contents of protein, fibre, vitamins (e.g. vitamins A and C) and minerals (e.g. K and Fe)^(5–7)^. In fact, an inverse association between HPF consumption and overall diet quality has been observed in many countries^(5–20)^.

Meanwhile, little evidence is available regarding HPF consumption in Japan. Although the Japanese dietary pattern is considered to be based on dishes and meals made from a variety of unprocessed or less processed foods^(21)^, HPF consumption may not be low, as Japan ranks 10th out of 80 countries in annual per capita retail sales of HPF^(22)^. To our knowledge, there have been only two studies on HPF consumption in Japan^(23,24)^. However, these studies have been conducted in a single prefecture in Japan, and HPF consumption in a diverse geographic population is unknown. Furthermore, despite the increasing consumption of alcoholic beverages and ready-made foods in Japan^(25,26)^, these items were excluded from the previous studies, which may have led to the misestimation of HPF consumption. Moreover, obesity and depression, which have been reported to be positively associated with HPF consumption^(3,4)^, are increasing in Japan^(27,28)^. Thus, HPF consumption by the Japanese should be carefully assessed in diverse geographic areas, including all the foods and beverages consumed.

The Food and Agricultural Organization recommends, in its guidelines for collecting information on food processing, distinguishing between food and dish items processed in industrial settings and those prepared by hand at home or in artisanal settings (e.g. street foods) and disaggregating recipes into their ingredients when possible^(29)^. However, the distinction between artisanal and industrial foods is ambiguous^(30)^, and the types of dishes broken down vary among studies^(20,31)^. Specifically, there is no consensus on whether to disaggregate dishes prepared away from home (e.g. ready-made dishes bought from supermarkets and restaurant meals). Previous studies have indicated that the discrepancy between classification methods may lead to a different conclusion regarding HPF consumption and its relationship to diet quality^(30,32)^.

Therefore, the present study aimed to assess HPF consumption and its association with diet quality in Japanese adults. Moreover, we compared the results obtained when dishes prepared away from home were disaggregated into ingredients before classification by processing levels with the results when they were classified without disaggregation. Such investigations would be useful to understand the differences in the estimates of HPF consumption by classification methods. Based on existing literature, we hypothesised that the energy contribution of HPF would be inversely associated with overall dietary quality and that differences in classification methods might affect estimates of HPF contribution.

## Methods

### Data source

In this cross-sectional study, we used dietary data from a nationwide survey conducted at single point in time between February and March 2013 in twenty study areas, consisting of twenty-three prefectures in Japan. The original purpose of the survey was to evaluate the amount and source of Na intake in Japanese adults. The details of the survey have been described elsewhere^(33)^. Briefly, 199 research dietitians working at separate welfare facilities invited their colleagues and family members of the colleagues to participate in the study. Approximately four apparently healthy subjects (two males and two females) were recruited from five 10-year age categories (20–29, 30–39, 40–49, 50–59 and 60–69 years) in each study area. Participation in the survey was limited to one participant per household. No participant was a dietitian or a medical professional, had received dietary therapy from a doctor or dietitian, had a history of educational hospitalisation for diabetes or was a pregnant or lactating woman. In total, 196 males and 196 females aged 20–69 years provided the dietary data. After excluding four participants with missing information on the variables of interest, 388 participants (196 males and 192 females) were included in the present analysis.

### Dietary assessment

Dietary data were obtained using a 4-day weighed dietary record (DR). The details of the DR are provided elsewhere^(33)^. Briefly, participants were asked to record all foods and beverages consumed on four non-consecutive days (three working days and one non-working day, excluding night-shift days and days before and after a night shift). Research dietitians explained how to keep the DR on the participants and requested them to weigh foods and beverages with a digital scale (KD-812WH) or measure with the spoon and cup provided.

The recording sheet included the following items: (i) dish names, (ii) whether dishes were prepared at home, away from home or other (foods eaten in a raw state, such as fresh fruits and vegetables), (iii) food names (ingredients included in dishes) and (iv) approximate amounts or measured weights of foods consumed. Participants were also asked to record the names of products and manufacturers for store-bought products and the names of menus and restaurants when they dined out. In addition, participants were asked to collect packaged food packages.

Recording sheets and packages were submitted to a research dietitian at each facility immediately after recording. The research dietitians reviewed the recording sheets as soon as possible and, if necessary, asked the participants to provide additional information to clarify the name or amount of food on the sheet. For packaged foods and dishes prepared away from home, each food ingredient and its consumed weight were estimated as precisely as possible based on the approximate amount of food, the website of the restaurant or manufacturer, ingredient labels, nutrition facts labels on food packages and cooking books. Each food item was assigned a food code from the Standard Tables of Food Composition in Japan (STFCJ)^(34)^ in a uniform procedure.

All foods items recorded in the column ‘food names’ were then classified into three categories by the research dietitian as follows: (1) ‘home-made’: foods cooked at home (e.g. rice cooked at home and bread baked at home); (2) ‘store-bought’: ingredients of dishes prepared away from home and foods processed by the manufacturer (e.g. ready-to-heat curry, processed meat and chocolate) and (3) ‘other’: unprocessed ingredients before cooking at home (e.g. fresh vegetables, meats, fish and milk) or seasonings added during home cooking or used at the table at home (e.g. mayonnaise used to make sandwiches). In addition to this classification, all food codes and weights were reconfirmed by two other research dietitians at the central office of this study.

### Classification of foods based on the degree of food processing

We used the framework developed by researchers at the University of North Carolina at Chapel Hill (UNC)^(1)^ to classify foods according to the level of processing. The UNC system, developed based on the most widely used classification system, NOVA^(21)^, provides enhanced definitions of food categories^(1)^ (online Supplementary Table 1). Although the UNC system classifies packaged food products with a barcode sold in the USA, it would be useful to classify unpackaged food products, as it provides broad and detailed examples of foods for each category, ranging from fresh vegetables (e.g. whole carrots) to refrigerated ready-made vegetable-based mixed dishes (e.g. coleslaw). A previous study showed that the UNC system had higher inter-rater reliability than the NOVA classification system^(35)^.

**Fig. 1:**
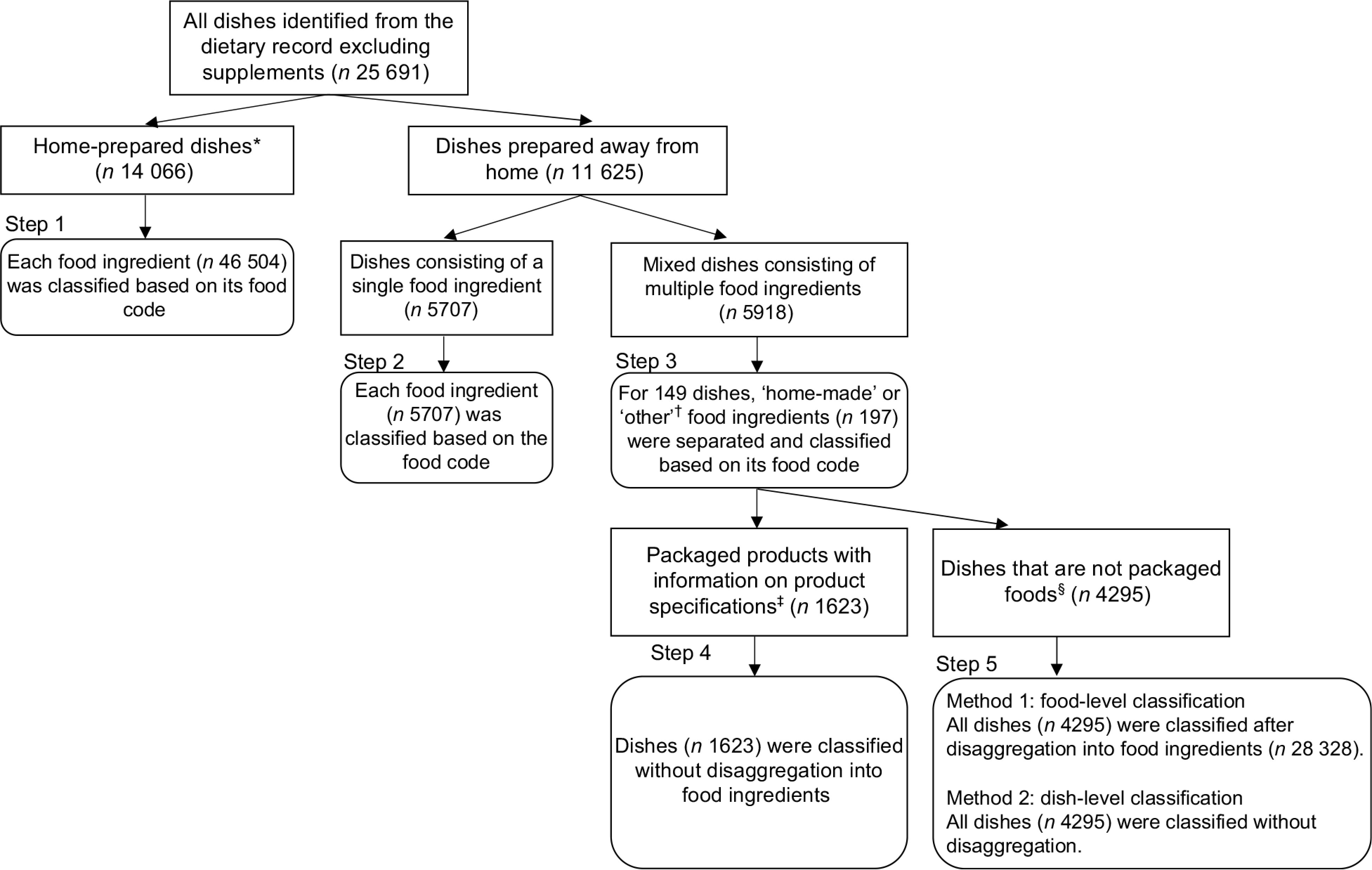
Flow chart of the classification of foods in the 4-day dietary record obtained from Japanese adults aged 20–69 years (*n* 388) in 2013

According to the UNC system, the author classified all foods (except for dietary supplements) in the DR into one of the four groups:(1) unprocessed and minimally processed, (2) basic processed, (3) moderately processed and (4) highly processed. We decided to break down home-prepared dishes into component ingredients before classification, but not to break down industrial packaged food products, according to the guidelines of the Food and Agricultural Organization^(29)^ and previous studies^(9,10,14,17,31)^. However, there is no consensus on whether dishes prepared away from home (e.g. ready-made dishes from the supermarket and restaurant meals) should be disaggregated. Therefore, we classified dishes prepared away from home at both the food level (classified after disaggregation into ingredients) and the dish level (classified without disaggregation). The food classification procedure is shown below and in Fig. 1.

- Step 1: Home-prepared dishes (including foods eaten raw) were disaggregated into component ingredients, each of which was classified based on its food code (e.g. miso, water, spinach and egg in miso soup cooked at home, fresh fruits and vegetables).
- Step 2: For ready-made dishes consisting of a single food ingredient, each food ingredient was classified based on its food code (e.g. black tea, black coffee and candy).
- Step 3: If dishes prepared away from home contained ‘home-made’ or ‘other’ food ingredients (mostly seasonings, such as soy sauce added to Chinese dumplings at home), that ingredient was individually classified based on its food code.
- Step 4: Packaged food products (i.e. dishes prepared away from home containing multiple food ingredients with the names of products, brands or manufacturers of packaged foods) were classified without disaggregation. For instance, we classified packaged sandwiches from convenience stores as single items.
- Step 5: Other dishes prepared away from home (e.g. dishes purchased from supermarkets, restaurant meals and other miscellaneous dish items without information identifying them as packaged food) were classified at two levels: 1) the food level: all items were classified after disaggregation into food ingredients or 2) the dish level: all items were classified without disaggregation.

In Steps 1–3 and the food-level classification in Step 5, each food item was classified based on the food code of the STFCJ^(34)^, considering whether each food item was categorised as ‘home-made’ or ‘store-bought’ by the research dietitian. For instance, among tea with food code 16 042 ‘oolong tea, infusion’, those considered ‘home-made’ were classified into the unprocessed and minimally processed category. By contrast, those considered ‘store-bought’ were classified into the basic processed category. For the dish-level classification in Step 5, we assumed that all dishes were ready-to-eat or ready-to-heat foods rather than frozen or shelf-stable foods.

### Calculation of diet quality scores

The mean daily intake of energy and nutrients over 4 days was calculated for each participant based on the weight and nutrient content of each food item using the STFCJ^(34)^. For foods with added sugar content not available in the STFCJ, added sugar values were calculated based on the same or similar food items in the 2011–2012 Food Patterns Equivalents Database^(36)^. We converted the teaspoon equivalents in the Food Patterns Equivalents Database into grams by multiplying by 4·2 (grams of added sugar per teaspoon).

We assessed the diet quality of each participant using the Healthy Eating Index-2015 (HEI-2015)^(37)^ and Nutrient-Rich Food Index 9·3 (NRF9·3)^(38)^. The usefulness of these indices has been verified in the Japanese population^(39)^. The HEI-2015 evaluates a set of foods on a 100-point scale for compliance with the 2015–2020 Dietary Guidelines for Americans^(40)^, with a higher score indicating better diet quality. The NRF9·3 is a composite measure of nutrient density calculated as the sum of the percentage of the reference daily value of nine qualifying nutrients minus the sum of the percentage of the reference daily values of three disqualifying nutrients. The maximum possible score was 900, with the higher NRF9·3 total score indicating a better diet quality. Details on the calculation of both indices are provided in Supplementary Text 1.

### Assessment of basic characteristics

Body weight (in 0·1 kg) and height (in 0·1 cm) were measured by research dietitians or medical workers using standardised procedures while the participant was in light clothes without shoes. BMI was calculated as body weight (kg) divided by the square of height (m^2^). In addition, information on sex, age, education level (junior high school or high school, vocational school or junior college, or university or graduate school), self-reported hours spent per day or week on six activities (walking, cycling, standing, running, exercise causing sweating and sleeping) and smoking status (never, past or current) was collected using a questionnaire. Physical activity (total metabolic equivalents, h/d) was calculated by summing the product of the self-reported hours spent per day on each activity during the preceding month and the corresponding metabolic equivalent value^(41,42)^.

### Data analysis

All analyses were conducted using the statistical software package SAS version 9.4 (SAS Institute Inc.). Two-sided *P*-values < 0·05 were considered statistically significant. First, all foods were categorised into food groups based on the similarity of nutrient composition or culinary use, mainly according to the STFCJ^(34)^. The contribution (%) of each food group to the total energy intake of HPFs over 4 days was calculated for the whole population.

Next, the mean daily dietary intake over 4 days was calculated for each participant and used for all subsequent analyses. We calculated the mean energy contribution (%) of each of the four processing categories using the dish- and food-level classifications for dishes prepared away from home. A paired *t* test was used to compare the mean energy contributions for each food category between the dish- and food-level classification. Pearson correlation coefficients were calculated to examine the associations between energy contributions from each processed food category when the dish- and food-level classifications were used. Pearson’s correlation coefficients were also computed to evaluate the association between energy contributions from different processed food categories within the same classification level.

In addition, the participants were divided into tertiles of the proportion of energy from HPF using both food- and dish-level classifications. We calculated the mean and sd or the number and percentage for each basic characteristic variable in each tertile of the proportion of energy from the HPF. Differences in basic characteristics across tertiles were assessed using the Mantel–Haenszel extension χ^2^ test for categorical variables (sex, education level and smoking status) and a linear trend test for continuous variables (age, height, body weight, BMI and physical activity). For linear regression, the median value in each tertile category of the energy contribution of the HPF was used as a continuous variable. Similarly, we calculated the average food group intake in each tertile group of the HPF energy contribution and tested the linear trend with increasing levels of food group intake using linear regression analysis. Finally, we calculated the adjusted means of the total and component scores of the HEI-2015 and NRF9·3 for each tertile of the energy contribution of the HPF. Potential confounding factors adjusted for were age, sex, BMI, physical activity, education level and smoking status^(9,13,15,19)^. We tested for linear trends in diet quality scores across tertiles by assigning the median value of the energy contribution of HPF for each group as a continuous variable.

## Results

### The contribution of each food group to energy intake from HPFs

**Fig. 2:**
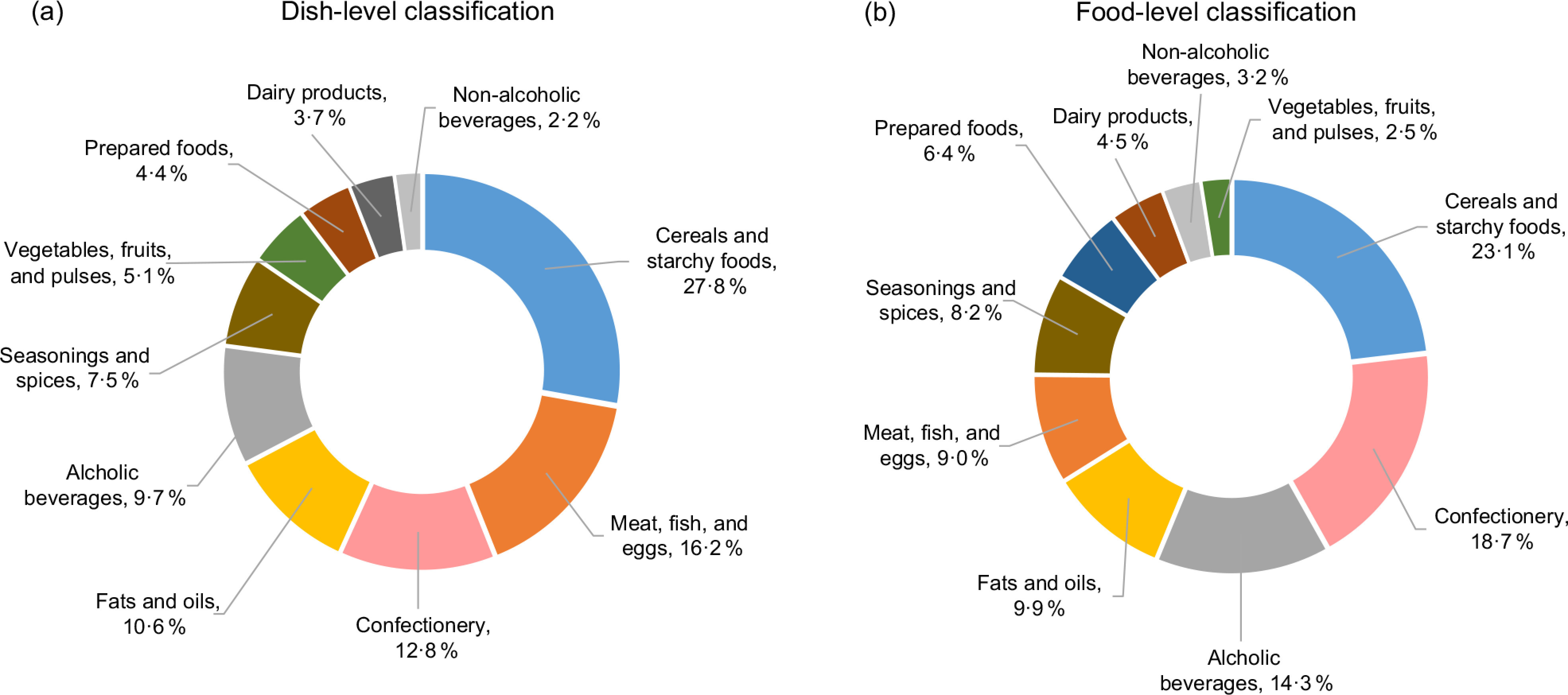
Relative contribution of each food group to the total energy intake of highly processed foods in the entire population (*n* 388). In the dish-level classification, dishes prepared away from home (e.g. ready-made dishes from the supermarket and restaurant meals) were classified by level of processing without recipe disaggregation. In contrast, they were classified after disaggregation into component ingredients in the food-level classification

The mean age of the study participants was 44·5 years (sd 13·3) and the mean BMI was 23·3 kg/m^2^ (sd 3·7). Figure 2 shows the relative contribution of each food group to energy intake from HPF in the entire population. When dishes prepared away from home were classified at the dish level, the main food groups contributing to total energy intake from HPF were cereals and starchy foods (27·8 %), followed by meat, fish and eggs (16·2 %), confectionery (12·8 %), fats and oils (10·6 %) and alcoholic beverages (9·7 %). These food groups were also ranked in the top five when the food-level classification was used, albeit in a different order: cereals and starchy foods (23·1 %), followed by confectionery (18·7 %), alcoholic beverages (14·3 %), fats and oils (9·9 %) and meat, fish and eggs (9·0 %).

### The energy contribution of foods classified by the level of food processing

Table 1 shows the descriptive statistics of the energy contribution of foods classified by the level of food processing. The energy contribution of HPF was significantly higher when dishes prepared away from home were classified at the dish level than the food level (48·3 % *v*. 32·9%, *P* < 0·0001). Conversely, the dish-level classification provided significantly lower estimates of energy contributions than the food-level classification for the other food categories, that is, unprocessed/minimally processed foods, basic processed foods and moderately processed foods (*P* < 0·0001 for all). The energy contributions from foods in each processing category between the dish- and food-level classifications were strongly correlated (*r* = 0·71–0·84). Moreover, for both dish- and food-level classifications, the energy contribution of HPF was significantly inversely correlated with unprocessed/minimally processed foods (r = –0·71 and –0·55, respectively) and basic processed foods (r = –0·78 and –0·70, respectively).

**Table 1:** Descriptive statistics of energy contribution (%) from foods classified by level of food processing; Japanese adults aged 20–69 years (*n* 388), 2013

			Dish-level classification*				Food-level classification†			
	Mean‡	sd	UPF	BPF	MPF	HPF	UPF	BPF	MPF	HPF
Dish-level classification*										
UPF	16·9	8·3	1·00	0·17	0·21	−0·71	0·79	−0·06	0·01	−0·46
BPF	29·8	10·9		1·00	−0·04	−0·78	−0·04	0·84	−0·19	−0·61
MPF	5·0	3·6			1·00	−0·31	0·07	−0·13	0·76	−0·20
HPF	48·3	15·6				1·00	−0·41	−0·53	−0·04	0·71
Food-level classification†										
UPF	23·4	7·3					1·00	−0·10	−0·02	−0·55
BPF	36·8	9·6						1·00	−0·20	−0·70
MPF	6·9	4·1							1·00	−0·18
HPF	32·9	11·5								1·00

UPF, unprocessed or minimally processed foods; BPF, basic processed foods; MPF, moderately processed foods; HPF, highly processed foods.

* Dishes prepared away from home (e.g. ready-made dishes from the supermarket and restaurant meals) were classified without recipe disaggregation.

† Dishes prepared away from home (e.g. ready-made dishes from the supermarket and restaurant meals) were classified after disaggregation into food ingredients.

‡ *P* < 0·0001 for all differences in the energy contribution of each food group between the dish- and food-based classification methods (paired *t* test).

### Participant characteristics and energy and nutrient intake in relation to HPF consumption

Participant characteristics according to tertiles of the energy contribution of the HPF are shown in Table 2. In both classifications, the higher tertiles of energy contribution of HPF included younger participants and current smokers. Table 3 shows the energy and food group intake by tertiles of the energy contribution of the HPF. The results were similar for both the classifications. The mean energy intake did not differ across tertile groups, whereas the mean intake of some food groups differed. For instance, participants in higher tertiles had lower intakes of cereals and starchy foods; fruits, vegetables and pulses; meat, fish and eggs; and higher intakes of confectioneries; alcoholic beverages; fats and oils and seasoning and spices.

**Table 2:** Participant characteristics by tertiles (T) of energy contribution of highly processed foods*; Japanese adults aged 20–69 years (*n* 388), 2013

			Dish-level classification†							Food-level classification‡						
	All		T1		T2		T3			T1		T2		T3		
	(*n* 388)		(*n* 129)		(*n* 130)		(*n* 129)		*P*_trend_ §	(*n* 129)		(*n* 130)		(*n* 129)		*P*_trend_ §
Age (years)	44·5	13·3	48·2	13·4	45·9	12·9	39·4	12·1	< 0·0001	47·6	14·2	44·7	12·8	41·3	12·2	0·0001
Height (cm)	164·0	8·4	163·7	8·2	162·4	8·7	165·9	8·1	0·03	163·0	8·2	164·0	9·1	164·9	7·9	0·07
Body weight (kg)	62·9	12·7	63·1	13·0	61·7	13·7	64·0	11·2	0·57	61·5	12·2	64·4	13·9	62·8	11·7	0·51
BMI (kg/m^2^)	23·3	3·7	23·4	3·9	23·2	3·8	23·2	3·1	0·51	23·0	3·7	23·8	3·8	23·0	3·4	0·77
Physical activity (MET × h)	37·3	5·7	38·0	5·7	37·0	5·2	36·8	6·0	0·11	37·8	5·8	36·7	5·6	37·3	5·5	0·54
Female (*n*, %)	192	49·5	64	49·6	73	56·2	55	42·6	0·26	63	48·8	64	49·2	65	50·4	0·80
Education level (*n*, %)									0·12							0·14
Junior or senior high school	110	28·4	44	34·1	38	29·2	28	21·7		44	34·1	36	27·7	30	23·3	
Vocational school or junior college	144	37·1	41	31·8	50	38·5	53	41·1		46	35·7	43	33·1	55	42·6	
University or graduate school	134	34·5	44	34·1	42	32·3	48	37·2		39	30·2	51	39·2	44	34·1	
Smoking status (*n*, %)									0·002							0·000
Never smoker	217	55·9	79	61·2	78	60·0	60	46·5		81	62·8	76	58·5	60	46·5	
Past smoker	71	18·3	27	20·9	21	16·2	23	17·8		26	20·2	24	18·5	21	16·3	
Current smoker	100	25·8	23	17·8	31	23·9	46	35·7		22	17·1	30	23·1	48	37·2	

MET, metabolic equivalents.

* Values are means and standard deviations for continuous variables unless otherwise indicated.

† Dishes prepared away from home (e.g. ready-made dishes from the supermarket and restaurant meals) were classified without recipe disaggregation.

‡ Dishes prepared away from home (e.g. ready-made dishes from the supermarket and restaurant meals) were classified after disaggregation into food ingredients.

§ Derived from a Mantel–Haenszel extension χ^2^ test for categorical variables and linear trend test for continuous variables. For linear regression, the median value in each tertile category of the energy contribution of highly processed foods was used as a continuous variable. The median (range) energy contribution (%) of highly processed foods per tertile were as follows: 1st = 32·5 (14·8–40·9), 2nd = 47·7 (41·2–54·2) and 3rd = 62·7 (54·5–100) for the dish-level classification method; and 1st = 21·9 (9·6–26·7), 2nd = 31·1 (26·9–37·4) and 3rd = 43·6 (37·5–79·6) for the food-level classification method.

**Table 3:** Intakes of energy (kJ/d) and food groups (g/d) according to the tertiles (T) of the energy contribution of highly processed foods; Japanese adults aged 20–69 years (*n* 388), 2013

			Dish-level classification*							Food-level classification†						
	All		T1		T2		T3			T1		T2		T3		
	(*n* 388)		(*n* 129)		(*n* 130)		(*n* 129)			(*n* 129)		(*n* 130)		(*n* 129)		
	Mean	s d	Mean‡	se	Mean‡	se	Mean‡	se	*P*_trend_ §	Mean‡	se	Mean‡	se	Mean‡	se	*P*_trend_ §
Energy intake (kJ/d)	8912	2038	8812	162	9005	158	8919	165	0·64	8680	161	9058	158	8997	162	0·21
Cereals and starchy foods	500	155	545	11	498	11	457	12	< 0·0001	542	11	506	11	452	11	< 0·0001
White rice	324	145	386	11	322	11	266	11	< 0·0001	374	11	333	11	266	11	< 0·0001
Brown rice	3	27	7	2	3	2	1	2	0·11	5	2	5	2	1	2	0·29
White bread	34	30	26	3	36	3	39	3	0·001	26	3	33	3	42	3	< 0·0001
Wholegrain bread	1	5	1	0	0	0	1	0	0·93	1	0	0	0	1	0	0·97
Noodles	76	69	67	6	70	6	91	6	0·01	72	6	73	6	84	6	0·13
Other grain products	18	21	15	2	19	2	20	2	0·03	19	2	16	2	19	2	0·95
Potatoes	44	35	45	3	48	3	39	3	0·22	46	3	46	3	40	3	0·17
Fruits, vegetables and pulses	405	190	447	15	404	15	363	15	0·0001	461	15	401	15	352	15	< 0·0001
Fruit	62	70	67	5	56	5	63	6	0·56	67	5	62	5	56	5	0·18
Total vegetable	259	121	290	10	269	10	219	10	< 0·0001	296	10	261	10	221	10	< 0·0001
Pulses	60	53	66	5	58	5	55	5	0·14	74	5	56	5	50	5	0·0006
Fruit and vegetable juice	24	54	25	5	22	5	26	5	0·88	25	5	22	5	25	5	0·95
Meat, fish and eggs	198	75	204	6	206	6	184	6	0·04	209	6	208	6	176	6	0·0001
Meat	91	53	93	5	91	4	90	5	0·60	95	4	96	4	83	4	0·05
Fish	69	46	70	4	75	4	62	4	0·20	74	4	74	4	59	4	0·006
Eggs	38	22	41	2	40	2	33	2	0·005	40	2	39	2	34	2	0·03
Dairy products	105	96	108	9	111	8	97	9	0·40	106	9	104	8	107	9	0·92
Ice cream	4	11	4	1	3	1	4	1	0·77	2	1	5	1	4	1	0·25
Cheese	4	6	3	1	4	1	4	1	0·27	3	1	4	1	4	1	0·37
Milk	64	81	72	7	70	7	52	7	0·07	70	7	60	7	63	7	0·51
Yoghurt	26	41	25	4	27	3	26	4	0·91	27	4	25	3	26	4	0·78
Other dairy products	7	26	4	2	7	2	11	2	0·03	3	2	9	2	10	2	0·03
Confectioneries	38	34	28	3	38	3	48	3	< 0·0001	23	3	41	3	50	3	< 0·0001
Sweet buns	5	12	3	1	6	1	6	1	0·04	2	1	4	1	8	1	0·0004
Salty snacks	4	7	4	1	4	1	5	1	0·17	3	1	5	1	5	1	0·01
Chocolates, biscuits and cookies	8	11	6	1	7	1	10	1	0·008	5	1	8	1	10	1	0·0007
Other confectionaries	21	25	15	2	22	2	27	2	0·0003	13	2	24	2	27	2	< 0·0001
Alcoholic beverages	137	257	77	21	140	21	193	21	0·0002	49	20	123	20	239	20	< 0·0001
Beer	95	227	52	19	89	19	145	20	0·001	32	19	81	18	173	19	< 0·0001
Wine	7	34	7	3	8	3	6	3	0·76	3	3	8	3	10	3	0·15
Japanese Sake	19	61	2	3	11	3	7	3	0·28	1	3	6	3	14	3	0·002
Shochu	7	34	9	5	22	5	26	5	0·03	7	5	18	5	33	5	0·0005
Other alcoholic beverages	9	20	6	2	10	2	10	2	0·13	6	2	11	2	9	2	0·30
Non-alcoholic beverages	1344	646	1314	58	1390	56	1328	59	0·84	1265	57	1360	56	1407	58	0·10
Sugar-sweetened beverages	40	88	25	8	40	8	56	8	0·006	17	8	38	7	65	8	< 0·0001
Tea	481	360	484	32	485	31	474	33	0·83	474	32	518	31	452	32	0·55
Coffee	310	397	287	36	345	35	298	36	0·79	264	35	306	35	361	35	0·06
Non-caloric beverages	2	15	3	1	4	1	0	1	0·17	1	1	3	1	1	1	0·92
Water	510	349	515	31	516	31	499	32	0·73	509	31	495	31	528	31	0·64
Fats and oils	21	10	19	1	23	1	22	1	0·004	20	1	22	1	23	1	0·03
Oils	10	6	9	1	10	1	10	1	0·28	10	1	10	1	10	1	0·86
Fats	4	4	3	0	4	0	5	0	0·0004	3	0	4	0	4	0	0·06
Mayonnaise and dressing	7	6	7	1	8	1	7	1	0·34	6	1	8	1	8	1	0·04
Seasonings and spices	128	84	114	7	132	7	137	8	0·03	141	7	125	7	117	7	0·03
Sugar	13	11	11	1	12	1	16	1	< 0·0001	12	1	14	1	14	1	0·09
Jam	2	6	2	1	2	0	2	1	0·84	2	1	3	0	2	1	0·69
Salt	2	1	2	0	2	0	2	0	0·57	2	0	2	0	2	0	0·25
Soy sauce	17	9	18	1	18	1	16	1	0·31	18	1	18	1	16	1	0·055
Other seasonings	94	80	82	7	99	7	101	7	0·07	108	7	89	7	84	7	0·03
Ready-made dishes	31	38	30	3	29	3	35	3	0·35	23	3	31	3	40	3	0·0008
Ready-to-eat dishes‖	22	36	20	3	20	3	26	3	0·14	14	3	22	3	31	3	0·0001
Pickles	9	14	11	1	8	1	9	1	0·24	10	1	9	1	9	1	0·61

* Dishes prepared away from home (e.g. ready-made dishes from the supermarket and restaurant meals) were classified without recipe disaggregation.

† Dishes prepared away from home (e.g. ready-made dishes from the supermarket and restaurant meals) were classified after disaggregation into food ingredients.

‡ Least square means adjusted for age, BMI, physical activity, sex, education level and smoking status.

§ Derived from linear regression using the median value in each tertile category of the energy contribution of highly processed food as a continuous variable. The median (range) energy contribution (%) of highly processed foods per tertile were as follows: 1st = 32·5 (14·8–40·9), 2nd = 47·7 (41·2–54·2) and 3rd = 62·7 (54·5–100) for the dish-level classification method; and 1st = 21·9 (9·6–26·7), 2nd = 31·1 (26·9–37·4) and 3rd = 43·6 (37·5–79·6) for the food-level classification method. The models were adjusted for age, BMI, physical activity, sex, education level and smoking status.

‖ Including ready-to-eat or ready-to-heat foods such as curry, Chinese dumplings, Hamburg steak, pilaf, meatballs and croquettes.

### Diet quality and HPF consumption

The mean (sd) scores of HEI-2015 and NRF9·3 scores were 51·1 (7·5) and 621·9 (116), respectively (Table 4). For both dish-level and food-level classifications, participants in higher tertiles of energy contribution of HPF had lower HEI-2015 total scores and component scores for total vegetables, greens and beans, total protein foods and added sugars and a higher score for refined grain. Moreover, participants in the higher tertiles had a lower component score of saturated fat only when the dish-level classification was used. Similarly, participants in higher tertiles of energy contribution of HPFs had lower NRF9·3 total scores and component scores for dietary fibre, vitamin A, vitamin C, vitamin D, Fe, K and Mg, and a higher score for added sugars, regardless of the classification levels. The component score for saturated fat was higher in participants in higher tertiles only when the dish-level classification was used.

**Table 4:** Diet quality scores according to the tertiles (T) of the energy contribution of highly processed foods; Japanese adults aged 20–69 years (*n* 388), 2013

			Dish-level classification*							Food-level classification†						
	All		T1		T2		T3			T1		T2		T3		
	(*n* 388)		(*n* 129)		(*n* 130)		(*n* 129)			(*n* 129)		(*n* 130)		(*n* 129)		
	Mean	sd	Mean‡	se	Mean‡	se	Mean‡	se	*P*_trend_ §	Mean‡	se	Mean‡	se	Mean‡	se	*P*_trend_ §
HEI-2015 (100)‖	51·1	7·5	52·4	0·6	51·2	0·6	49·6	0·6	0·002	52·2	0·6	51·1	0·6	49·8	0·6	0·007
Total fruits (5)	1·3	1·3	1·4	0·1	1·2	0·1	1·4	0·1	0·85	1·4	0·1	1·3	0·1	1·2	0·1	0·18
Whole fruits (5)	2·0	1·8	2·2	0·1	1·9	0·1	2·0	0·1	0·48	2·2	0·1	2·1	0·1	1·8	0·1	0·053
Total vegetables (5)	4·5	0·8	4·7	0·1	4·6	0·1	4·3	0·1	< 0·0001	4·7	0·1	4·7	0·1	4·3	0·1	< 0·0001
Greens and beans (5)	3·2	1·7	3·4	0·1	3·3	0·1	2·8	0·1	0·004	3·5	0·1	3·1	0·1	2·9	0·1	0·007
Whole grains (10)	0·5	1·4	0·6	0·1	0·4	0·1	0·6	0·1	0·84	0·6	0·1	0·5	0·1	0·5	0·1	0·66
Dairy (10)	1·9	1·6	1·9	0·1	2·0	0·1	1·9	0·1	0·88	1·9	0·1	1·9	0·1	2·0	0·1	0·46
Total protein foods (5)	4·7	0·6	4·8	0·1	4·8	0·1	4·6	0·1	0·01	4·8	0·1	4·8	0·1	4·5	0·1	< 0·0001
Seafood and plant proteins (5)	4·7	0·8	4·7	0·1	4·8	0·1	4·6	0·1	0·47	4·8	0·1	4·6	0·1	4·7	0·1	0·13
Fatty acids (10)¶	6·2	2·6	6·5	0·2	6·3	0·2	5·9	0·2	0·06	6·5	0·2	6·3	0·2	6·0	0·2	0·13
Refined grains (10)	1·4	2·1	1·1	0·2	1·4	0·2	1·8	0·2	0·01	1·0	0·2	1·4	0·2	2·0	0·2	0·0003
Sodium (10)	2·5	2·7	2·4	0·2	2·4	0·2	2·6	0·2	0·49	2·1	0·2	2·5	0·2	2·7	0·2	0·11
Saturated fats (10)	8·8	1·8	9·0	0·1	8·9	0·1	8·5	0·2	0·01	8·9	0·2	8·8	0·1	8·7	0·2	0·22
Added sugars (10)	9·2	1·4	9·7	0·1	9·3	0·1	8·6	0·1	< 0·0001	9·8	0·1	9·1	0·1	8·6	0·1	< 0·0001
NRF9·3 (900)**	621·9	116·3	666·3	8·9	633·4	8·7	565·9	9·1	< 0·0001	679·2	8·7	624·0	8·5	562·6	8·7	< 0·0001
Protein (100)	99·9	0·7	99·9	0·1	100·0	0·1	99·8	0·1	0·29	100·0	0·1	100·0	0·1	99·8	0·1	0·09
Dietary fibre (100)	76·0	16·7	79·8	1·4	75·8	1·3	72·5	1·4	0·0003	81·7	1·3	74·7	1·3	71·6	1·3	< 0·0001
Vitamin A (100)	65·3	20·9	68·4	1·8	67·3	1·7	60·2	1·8	0·002	69·8	1·8	67·5	1·7	58·6	1·8	< 0·0001
Vitamin C (100)	90·2	16·5	92·4	1·4	91·4	1·4	86·8	1·4	0·009	93·0	1·4	91·3	1·4	86·3	1·4	0·001
Vitamin D (100)	71·6	29·5	76·2	2·5	72·9	2·4	65·7	2·5	0·004	78·6	2·5	72·0	2·4	64·1	2·5	< 0·0001
Ca (100)	76·2	17·7	76·5	1·5	78·3	1·5	73·7	1·5	0·21	76·3	1·5	77·1	1·5	75·1	1·5	0·55
Fe (100)	92·1	13·4	93·1	0·9	93·1	0·9	90·0	1·0	0·03	93·9	0·9	93·2	0·9	89·1	0·9	0·0002
K (100)	92·7	10·4	94·2	0·9	93·9	0·9	90·0	0·9	0·002	94·8	0·9	93·5	0·9	89·7	0·9	< 0·0001
Mg (100)	89·6	12·2	91·3	1·0	90·9	1·0	86·7	1·0	0·003	94·0	1·0	89·3	1·0	85·6	1·0	< 0·0001
Added sugars (—)	50·3	63·9	26·4	5·4	46·4	5·2	78·1	5·4	< 0·0001	19·7	5·3	54·6	5·2	76·6	5·3	< 0·0001
Saturated fats (—)	22·3	24·7	18·6	2·1	21·5	2·0	26·8	2·1	0·008	20·3	2·1	22·3	2·1	24·4	2·1	0·17
Sodium (—)	59·0	35·0	60·5	3·1	62·1	3·0	54·5	3·1	0·20	63·0	3·1	57·8	3·0	56·3	3·1	0·15

HEI-2015, Healthy Eating Index-2015; NRF9·3, Nutrient-Rich Food Index 9·3.

* Dishes prepared away from home (e.g. ready-made dishes from the supermarket and restaurant meals) were classified without recipe disaggregation.

† Dishes prepared away from home (e.g. ready-made dishes from the supermarket and restaurant meals) were classified after disaggregation into food ingredients.

‡ Least square means adjusted for age, BMI, physical activity, sex, education level and smoking status.

§ Derived from linear regression using the median value in each tertile category of the energy contribution of highly processed food as a continuous variable. The median (range) energy contribution (%) of highly processed foods per tertile were as follows: 1st = 32·5 (14·8–40·9), 2nd = 47·7 (41·2–54·2) and 3rd = 62·7 (54·5–100) for the dish-level classification method; and 1st = 21·9 (9·6–26·7), 2nd = 31·1 (26·9–37·4) and 3rd = 43·6 (37·5–79·6) for the food-level classification method. The models were adjusted for age, BMI, physical activity, sex, education level and smoking status.

‖ Calculated as the sum of all component scores. The maximum scores are shown in parentheses. A higher score indicates higher diet quality.

¶ Defined as the ratio of the sum of polyunsaturated and MUFA to SFA.

** Calculated as the sum of scores for nine nutrients to encourage (i.e. protein, dietary fibre, vitamins A, C and D, Ca, Fe, K and Mg) minus the sum of scores for three nutrients to limit (i.e. added sugars, saturated fats and sodium). The maximum scores are shown in parentheses. For added sugars, saturated fats and sodium components, the maximum score was infinite, depending on the amount. A higher score indicates higher diet quality, except for added sugars, saturated fats and Na components, for which a higher score indicates a lower diet quality.

## Discussion

### Main findings

To the best of our knowledge, this is the first study to evaluate the consumption of HPF and their association with diet quality using data from a nationwide dietary survey in Japan. In Japan, dietary patterns have recently become continuously westernised^(26)^, and obesity (in males) and depression, both of which may be associated with HPF consumption^(3,4)^, are increasing^(27,28)^. Therefore, applying the classification method by the level of processing to foods consumed by the Japanese and thereby clarifying HPF consumption is important for future research on HPF and related health outcome, as well as for setting a public nutrition policy in Japan. In this study, we found that at least one-third of the total energy intake accounted for HPF, of which cereals and starchy foods were the main contributors. Participants in higher tertiles of the energy contribution of HPFs had lower total scores for the HEI-2015 and NRF9·3. Thus, as hypothesised, the dietary share of HPFs is inversely associated with the overall dietary quality. These results were consistent, regardless of whether dishes prepared away from home were broken down into ingredients before being categorised by the food processing level.

### Differences in food classification methods

There is no consensus regarding which dishes should be disaggregated into their ingredients before categorising them by processing levels^(30,32)^. For instance, the classification system used in the European Prospective Investigation into Cancer and Nutrition (EPIC) study breaks down all recipes^(20)^. On the other hand, a widely used classification system, NOVA, proposes to break down the recipes of dishes and meals made in traditional restaurants^(31)^. However, it is difficult to determine which restaurants are traditional and how they process their food^(30)^. Some previous studies disaggregated dishes prepared away from home, such as restaurant meals^(8,9)^, whereas others did not disaggregate^(15)^, excluded^(23)^ or did not mention how to treat them. A previous study has also highlighted inconsistencies in classification methods and indicated that the misclassification of foods may contribute to the misrepresentation of the consumption, nutrient profile and associated health outcomes of HPF^(32)^. In this study, the energy contribution of HPF was higher when dishes prepared away from home were classified without recipe disaggregation (48·3 %) than when they were disaggregated (32·9 %), whereas both estimates were strongly correlated. This result is reasonable because most dishes were categorised as HPF when classified as a single item. It should be noted, however, that there was about a 15 % difference in the estimated energy contribution from HPF depending on whether the food prepared outside the home was broken down or not.

### The energy contribution of HPFs

Our results showed that at least one-third of the total energy intake accounted for energy from the HPF. The energy contribution of HPF was similar to that in previous studies in Japan (38 %^(23)^ and 30 %^(24)^). Meanwhile, the energy contribution of HPF in this study was lower than those in Canada (54 %)^(5)^, the USA (58 %)^(18)^ and ten European countries attending the EPIC study (61—79 %)^(16,19,20)^, but higher than or equivalent to those in other areas or countries, such as Australia (39 %^(13)^), France (31 %)^(8,15)^, Mexico (30 %)^(7)^, Belgium (30 %)^(11)^, Chile (29 %)^(6)^, South Korea (25 %)^(12)^ and Brazil (20 %^(17)^). However, it should be noted that direct comparison of the results may be difficult because of the differences in classification systems or the choice of food items to disaggregate or omit.

### Participant characteristics and HPF consumption

Although associations between the energy contribution of HPFs and education level^(13,15)^ and sex^(19)^ have been reported previously, no association was observed in this study. However, the higher tertiles of the energy contribution of the HPF included younger participants and current smokers. This is consistent with the results of previous studies^(8–11,13,15,19)^. The inverse association between the energy contribution of HPF and age may be attributed to the fact that younger people tend to emphasise the convenience of food^(43)^, which is one of the facilitators of HPF consumption^(44)^.

### Diet quality and HPF consumption

^ ^39^^Consistent with previous studies^(5,6,8–10,12–18,23)^, the high-energy contribution of HPF was associated with a lower overall diet quality, regardless of the classification method of dishes prepared away from home. This may be due to the nutritional quality of HPF itself, food combinations, or dietary patterns in relation to HPF consumption. In addition, participants with higher energy intake from HPF had unfavourable intakes of total vegetables, greens and beans, total protein foods, added sugars, suggesting that increased HPF consumption is associated with a decrease in healthy food intake and an increase in unhealthy food intake. This finding is consistent with the results of previous studies^(9,14,19)^. However, contrary to a previous study in the USA^(14)^, refined grain intake was more favourable in the higher tertile group for the energy contribution of HPF. This is possibly due to the decrease in white rice in the higher tertile groups in this study.

Similar to a previous study^(15)^, participants with higher energy intake from HPF had unfavourable intakes of dietary fibre, vitamins, minerals and added or free sugars. However, in HEI-2015 and NRF9·3, the component score of sodium was not associated with the energy contribution of HPFs and was relatively low in all groups. This may be because unfavourable Na intake may be caused not only by HPF consumption, but also by other non-HPF sources, such as salt added to meals. Salt as a seasoning, which is categorised as a basic processed food, is the top contributor to sodium intake in this population^(28)^. Previous studies have reported inconsistent results regarding the association between the energy contribution of HPF and inadequate Na intake^(6,14,15)^ or Na density^(5,6,9,12,14–16,18)^. This may be explained by differences in the types or sodium contents of HPF consumed, eating habits in each country or food classification systems used. Indeed, the top food group contributing to the total energy intake from HPF differed among countries: soft and fruit drinks^(9)^ or fast food and ready-to-eat dishes^(5)^ in Canada, carbonated soft drinks in Chile^(6)^, processed meat in Belgium^(11)^, cookies and sweets in Brazil^(17)^, cookies, pastries and sweet bread in Mexico^(7)^, ready-to-eat meals in France^(15)^ and cereals and starchy foods (e.g. rice, bread and noodles) in Japan^(24)^, South Korea^(12)^, the USA^(18)^, the UK^(16)^ and ten countries participating in the EPIC study^(20)^.

### Implications for public health nutrition research and practice

This study’s findings have several implications for future research and practice. Given that the energy contribution of HPF is associated with low intake of unprocessed/minimally processed foods and low diet quality, reducing HPF may lead to an increase in non-HPF foods with a better nutritional profile^(5–7)^. The concept of HPF has been incorporated into official guidelines in several countries, including Brazil and Canada^(45)^. The American Heart Association also recommends choosing minimally processed foods over HPF^(46)^. However, some processed foods provide key nutrients, such as potassium and vitamin C, and have beneficial effects on food availability, convenience and safety^(47)^. Thus, uniformly reducing all HPF in heterogeneous foods may not be an appropriate public health goal^(48)^. Therefore, consideration is necessary about which HPF should be reduced or reformulated to improve diet quality. Moreover, the differences in HPF consumption among participants’ characteristics or eating occasions may provide valuable information on efficient intervention strategies to promote healthy eating habits. In addition, since the estimates of HPF contribution differ widely depending on whether dishes prepared away from home are disaggregated, it is necessary to confirm the process of food classification when comparing the results across studies. In addition, future research should clearly explain what types of foods are broken down and could benefit from the standard guidelines for recipe disaggregation.

### Strengths and limitations

The strength of this study is the use of 4-day DR obtained from a nationwide sample of Japanese adults. For the purpose of the survey, detailed information on foods was collected in the DR, such as the names of products, manufacturers and menus, as well as whether the food was prepared at home, away from home, or elsewhere. This allowed for the detailed classification of foods by processing level. In addition, comparing the results using two different classification methods for foods prepared away from home would be helpful in future studies on how to classify processed foods.

However, this study had some limitations. First, the participants voluntarily participated in the study and most worked at welfare facilities; therefore, the generalisability of our results may be limited. The proportions of current smokers and graduates from university or graduate school in this study were higher than those in the national survey (19·3 %^(49)^ and 19·9 %^(50)^, respectively). Nevertheless, participants were selected from all over Japan, and their mean height, weight and BMI did not materially differ from those of the general adult population (160 cm, 58·8 kg and 22·9 kg/m^2^ in 2013, respectively^(49)^). Second, the UNC system may be suboptimal for classifying foods sold in Japan because it was developed using the US food supply. Nevertheless, the UNC system provides detailed definitions and examples for foods in each processing category to help classify a variety of foods and has been applied to several countries outside the US, such as Portugal^(51)^ and Spain^(52)^. Third, the food classification was conducted by a single author and was not double-checked. Although the UNC system was found to have the highest inter-rater reliability among the three processing classification systems (including NOVA) used in the USA^(35)^, some foods may have been misclassified in this study. Moreover, the description of some foods and beverages in the DR may not have been sufficient to correctly classify foods, which could have resulted in inaccurate categorisation of, for example, packaged food products. Fourth, disaggregating dishes into food ingredients may lead to a misestimation of the type or amount of ingredients. To estimate the type or amount of food items as accurately as possible, we referred to various information on the food item, including the approximate total amounts, the website of the restaurant or manufacturer, ingredient and nutrition facts labels and typical recipes. Fifth, the classification process was determined based on the format of the recording sheets used in this study, which cannot be applied in other studies. Nevertheless, the methodology demonstrated in this study would be informative for future studies on processed foods, as detailed descriptions of the classification process, particularly for foods away from home, have not always been provided in previous studies. Finally, self-reported dietary data are subject to social desirability bias^(53)^, which may lead to a lower intake of unhealthy foods, potentially underestimating the contribution of HPF.

### Conclusion

HPF accounted for at least one-third of the total energy intake among Japanese adults. Moreover, the higher energy contribution of HPFs was associated with lower diet quality, regardless of whether dishes prepared away from home were broken down into ingredients before being categorised by the food processing level. Therefore, decreasing the consumption of HPF would potentially be an effective public strategy to improve the diet quality in Japanese adults. Further research is needed to develop successful policies and programmes to improve the diet quality related to HPF reduction in the Japanese context.

## Supplementary material

For supplementary material accompanying this paper visit https://doi.org/10.1017/S1368980023000721. click here to view supplementary material
