# Supplementary material for: Consumption of highly processed foods in relation to overall diet quality among Japanese adults: a nationwide study
Source: Public Health Nutr. 2023 Apr 24;26(9):1784–97. doi: 10.1017/S1368980023000721 (PMC10478055; doi:10.1017/S1368980023000721)
Supplement: Supplementary file 1 [file S1368980023000721sup001.pdf]

Supplementary Materials

Supplementary Table 1. The classification system developed by researchers at the University of North Carolina at Chapel Hill (UNC)[1].

Category	Subcategory	Definition	Example
Unprocessed/minimally processed*	---	Single-ingredient foods with no or very slight modifications that do not change inherent properties of the food as found in its natural form	Fresh plain milk, fresh, frozen, or dried plain fruit, eggs, brown rice, honey, herbs, spices
Basic processed*	Processed basic ingredients	Single isolated food components obtained by extraction or purification using physical or chemical processes that change inherent properties of the food	Unsweetened fruit juice not from concentrate, egg whites, whole-grain flour, oil, unsalted butter, sugar
	Processed for basic preservation or precooking	Single minimally processed foods modified by physical or chemical processes for the purpose of preservation or precooking but remaining as single foods	Unsweetened fruit juice from concentrate or frozen concentrate, unsweetened/unflavoured canned fruit, refined-grain pasta, sour cream, plain yoghurt
Moderately processed	Moderately processed for flavour	Single minimally or moderately processed foods with addition of flavour additives for the purpose of enhancing flavour; directly recognizable as original plant/ animal source	Sweetened/flavoured fruit or vegetable juice, sweetened/flavoured hot cereal, cheese, sweetened/flavoured yoghurt, salted butter
	Moderately processed grain products	Grain products made from whole-grain flour with water, salt, and/or yeast	Whole-grain breads, tortillas, crackers, or ready-to-eat cereals with no added sugar or fat
Highly processed	Highly processed ingredients	Multi-ingredient industrially formulated mixtures processed to the extent that they are no longer recognizable as their original plant/animal source and consumed as additions (condiments, dips, sauces, toppings, or ingredients in mixed dishes)	Tomato sauce, jelly, bread crumb with refined grains or added sugar/fat., margarine; mayonnaise, salad dressing, artificial sweetener, ketchup
	Highly processed stand-alone	Multi-ingredient industrially formulated mixtures processed to the extent that they are no longer recognizable as their original plant/animal source and not typically consumed as additions	Soda, alcohol, fruit drinks, sports drinks, onion rings, restructured potato chips, sausage, bread with refined grains or added sugar/fat, ice cream

*Unprocessed/minimally processed foods and basic processed were grouped as 'less processed' in the UNC system.

Reference

1. Poti JM, Mendez MA, Ng SW *et al* . (2015) Is the degree of food processing and convenience linked with the nutritional quality of foods purchased by US households? Am J Clin Nutr 101, 1251-1262.

Supplementary Text 1. Details on the calculation of diet quality scores

Healthy Eating Index 2015

The HEI-2015 evaluates a set of foods on a 100-point scale for compliance with the 2015–2020 Dietary Guidelines for Americans[1], with a higher score indicating better diet quality. The nine adequacy components (maximum score) are total fruits (5), whole fruits (5), total vegetables (5), greens and beans (5), wholegrains (5), dairy (10), total protein (5), seafood and plant protein (5), and fatty acids (the ratio of the sum of polyunsaturated and monounsaturated fatty acids to saturated fatty acids) (10); the four moderation components are refined grains (10), sodium (10), added sugars (10), and saturated fats (10). HEI-2015 total and component scores were calculated based on energy-adjusted values (except for fatty acids) of daily dietary intake for each participant, namely amount/4184 kJ of energy or percentage of energy, using the 2011–2012 Food Patterns Equivalents Database[2].

Nutrient-Rich Food Index 9.3

The NRF9.3 is a composite measure of nutrient density calculated as the sum of the percentage of the reference daily values (RDVs) of nine qualifying nutrients (protein, dietary fibre, vitamin A, vitamin C, vitamin D, calcium, iron, potassium, and magnesium) minus the sum of the percentage of the RDVs of three disqualifying nutrients (added sugars, saturated fats, and sodium). The RDV percentage of each qualifying nutrient was discarded at 100, as a high intake of one nutrient could not compensate for a low intake of another. Therefore, the maximum possible score was 900. We determined RDVs for sex and age categories based on the Dietary Reference Intakes (DRIs) for Japanese 2020[3], with the exception of added sugar. We used the Recommended Dietary Allowance for protein, vitamins A and C, calcium, iron, and magnesium; the tentative dietary goal for preventing lifestyle-related diseases for dietary fibre, potassium, saturated fats, and sodium; and the adequate intake of vitamin D. For added sugar, because of the low intake level[4] and no recommendation for added sugar intake in Japan, we used the conditional recommendation advocated by the World Health Organization (i.e., an upper limit of 5% of energy)[5]. For added sugar and saturated fats (RDVs were calculated as a percentage of energy), the energy intake was determined from the Estimated Energy Requirement for a moderate level of physical activity in the DRIs. We calculated NRF9.3 total and component scores based on daily nutrient intake for each participant, which was adjusted for the energy intake by the density method and then normalised for the sex- and age-specific Estimated Energy Requirement for a moderate physical activity level (from DRIs) and expressed as a percentage of the RDV.

References

1. U.S. Department of Health and Human Services and U.S. Department of Agriculture. 2015–2020 Dietary Guidelines for Americans. 8th Edition. https://health.gov/our-work/food-nutrition/2015-2020-dietary-guidelines/guidelines/ (accessed June 2022)
2. Bowman SA, Clemens JC, Thoerig RC *et al.* Food Patterns Equivalents Database 2011–2012: methodology and user guide. https://www.ars.usda.gov/northeast-area/beltsville-md-nrc/beltsville-human-nutrition-research-center/food-surveys-research-group/ (accessed June 2022)
3. Ministry of Health, Labour and Welfare, Japan. Dietary Reference Intakes for Japanese. 2020. https://www.mhlw.go.jp/content/10904750/000586553.pdf (accessed May 2022)(in Japanese)
4. Fujiwara A, Murakami K, Asakura K *et al.* (2018) Estimation of starch and sugar intake in a Japanese population based on a newly developed food composition database. *Nutrients* 10, 1474.
5. World Health Organization (2015) Guideline sugars intake for adults and children. https://www.who.int/publications/i/item/9789241549028 (accessed June 2022)
